# Development of a prototype clinical decision support tool for osteoporosis disease management: a qualitative study of focus groups

**DOI:** 10.1186/1472-6947-10-40

**Published:** 2010-07-22

**Authors:** Monika Kastner, Jamy Li, Danielle Lottridge, Christine Marquez, David Newton, Sharon E Straus

**Affiliations:** 1 Department of Health Policy, Management and Evaluation, Faculty of Medicine, University of Toronto, Health Sciences Building, 155 College Street, Suite 425, Toronto, ON, M5T 3M6, Canada; 2 Department of Mechanical and Industrial Engineering, University of Toronto, 5 King's College Road, Toronto, ON, M5S 3G8, Canada; 3 Li Ka Shing Knowledge Institute of St. Michael's hospital, University of Toronto, 30 Bond Street, Toronto, ON, M5B 1W8, Canada

## Abstract

**Background:**

Osteoporosis affects over 200 million people worldwide, and represents a significant cost burden. Although guidelines are available for best practice in osteoporosis, evidence indicates that patients are not receiving appropriate diagnostic testing or treatment according to guidelines. The use of clinical decision support systems (CDSSs) may be one solution because they can facilitate knowledge translation by providing high-quality evidence at the point of care. Findings from a systematic review of osteoporosis interventions and consultation with clinical and human factors engineering experts were used to develop a conceptual model of an osteoporosis tool. We conducted a qualitative study of focus groups to better understand physicians' perceptions of CDSSs and to transform the conceptual osteoporosis tool into a functional prototype that can support clinical decision making in osteoporosis disease management at the point of care.

**Methods:**

The conceptual design of the osteoporosis tool was tested in 4 progressive focus groups with family physicians and general internists. An iterative strategy was used to qualitatively explore the experiences of physicians with CDSSs; and to find out what features, functions, and evidence should be included in a working prototype. Focus groups were conducted using a semi-structured interview guide using an iterative process where results of the first focus group informed changes to the questions for subsequent focus groups and to the conceptual tool design. Transcripts were transcribed verbatim and analyzed using grounded theory methodology.

**Results:**

Of the 3 broad categories of themes that were identified, major barriers related to the accuracy and feasibility of extracting bone mineral density test results and medications from the risk assessment questionnaire; using an electronic input device such as a Tablet PC in the waiting room; and the importance of including well-balanced information in the patient education component of the osteoporosis tool. Suggestions for modifying the tool included the addition of a percentile graph showing patients' 10-year risk for osteoporosis or fractures, and ensuring that the tool takes no more than 5 minutes to complete.

**Conclusions:**

Focus group data revealed the facilitators and barriers to using the osteoporosis tool at the point of care so that it can be optimized to aid physicians in their clinical decision making.

## Background

Osteoporosis is a major public health concern, affecting over 200 million people worldwide [1], an estimated 10 million in the US [2], 4 million in the UK [3], and 1.4 million in Canada [4,5]. Without effective osteoporosis prevention and treatment, the burden of treating fractures in Canada is projected to reach $32.5 billion by the year 2018 [5,6], and $25.3 billion per year in the US by the year 2025 [4]. This is further compounded by the increasing proportion of people aged 65 and older, which will likely lead to increased numbers of people who will suffer from osteoporosis [6-8]. Fragility fractures are the clinical consequence of osteoporosis, and hip fractures have the most devastating prognosis [9,10], as they can significantly impair quality of life, physical function and social interaction, and can lead to admission to long-term care [10,11]. Although guidelines are available for best practice in osteoporosis [12-14], evidence indicates that patients are not receiving appropriate diagnostic testing [15,16] or treatment [17] according to guidelines. This evidence-to-care gap highlights the need for better knowledge translation (KT) strategies [18]. The use of clinical decision support systems (CDSSs) may be one solution because they can facilitate KT by providing high-quality evidence at the point of care. CDSSs can also promote disease management by generating patient-specific assessments or recommendations for clinicians through the input of patient data in a computer with a use of software algorithms that can match pieces of information from a knowledge database [19-22].

We conducted a systematic review of randomized controlled trials (RCTs) to identify and describe the effectiveness of tools that support clinical decision-making in osteoporosis disease management [23]. Findings from the review indicated that few osteoporosis CDSSs exist, and most of the 13 included studies evaluating these tools did not incorporate all 3 components of disease management (i.e. risk assessment, diagnosis, and treatment). However, interventions with multiple components (e.g. those that include reminders and education) and multiple targets (e.g. physicians and patients) appear more promising for increasing appropriate bone mineral density (BMD) testing and prescription of osteoporosis medications such as bisphosphonates than single-component or single-target interventions [23]. This is consistent with a more recent study, which found that compared with usual care, a multi-component intervention targeted to family physicians and patients increased BMD testing and prescription of osteoporosis medications in postmenopausal women with a wrist fracture [24].

**Figure 1:**
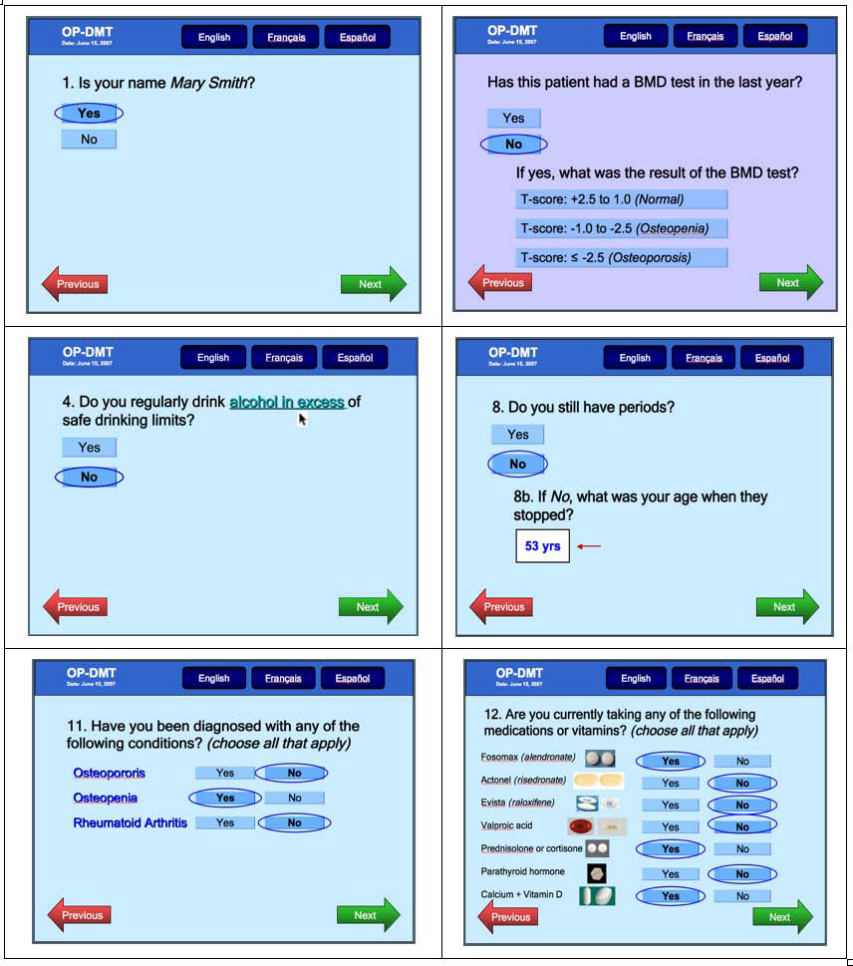
**Selected screen shots of the Risk Assessment Questionnaire (RAQ)**.

**Figure 2:**
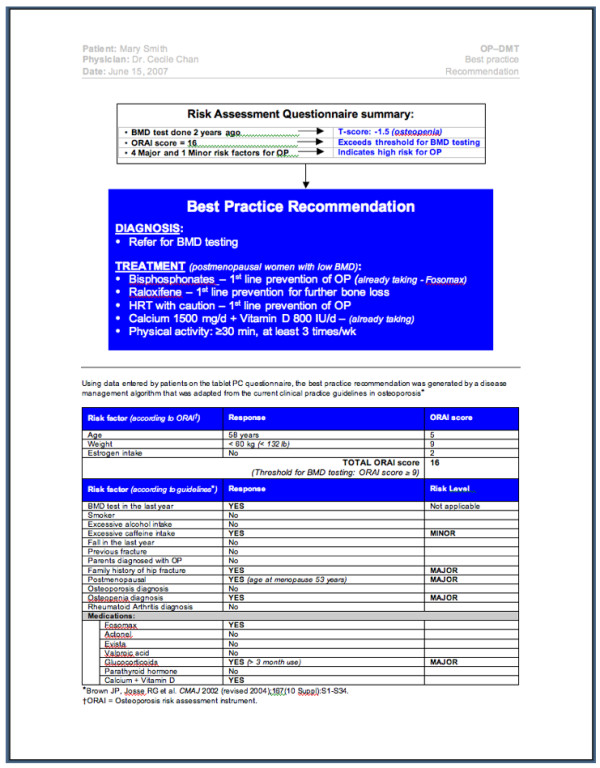
**Screen shot of the Best Practice Recommendation Prompt (BestPROMPT) sheet**.

**Figure 3:**
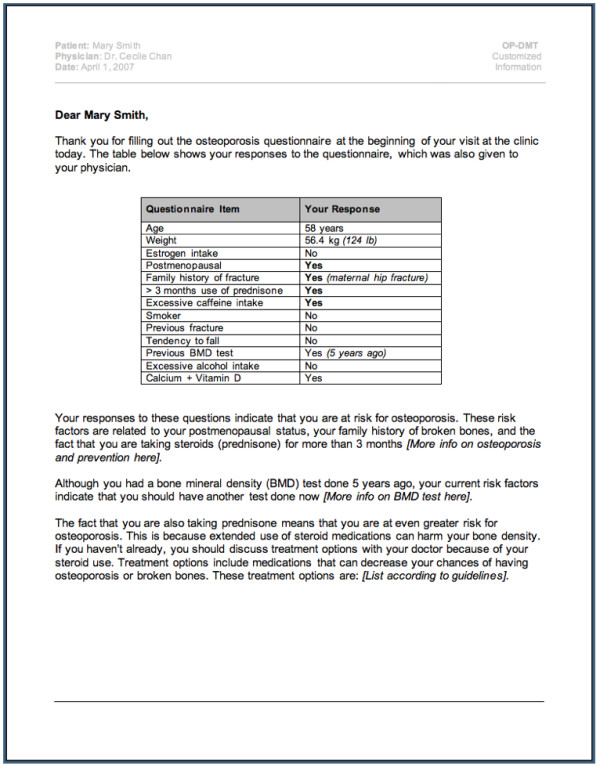
**Screen shot of the Customized Osteoporosis Education (COPE) sheet**.

Findings from our systematic review also highlighted the need to develop and rigorously evaluate an osteoporosis tool that can be used by physicians and patients at the point of care. Currently, FRAX^® ^is the only widely available electronic tool that can be used to assess a patient's 10-year probability of a fracture http://www.shef.ac.uk/FRAX[25]. However, this tool is not designed to assess all aspects of osteoporosis disease management; specifically, it does not provide customized, evidence-based osteoporosis diagnosis and treatment recommendations. We developed a conceptual model of an osteoporosis tool using systematic review findings and expert input from clinicians, information technologists and human factors engineers. The conceptual model was designed to incorporate all 3 aspects of disease management (i.e. risk assessment, diagnosis, and treatment), to target both physicians and patients, and to include 3 components. The first component of the osteoporosis tool is an electronic risk assessment questionnaire (RAQ) consisting of questions to assess osteoporosis risk as outlined by current practice guidelines [11]. The RAQ is designed so it can be completed on a Tablet PC by eligible patients (men ≥ women ≥ 50 years of age) in a clinic waiting room (Please see selected screen shots of the RAQ in Figure 1). Using a decision algorithm programmed into the Tablet PC, RAQ responses will then be processed, and two outputs generated (one for the physician and one for the patient), representing the second and third components of the osteoporosis tool. The second component of the tool is a paper-based, best practice recommendation prompt (BestPROMPT), which is a one-page sheet summarizing the patient's RAQ responses, a section providing appropriate osteoporosis disease management recommendations (e.g. to initiate bone mineral density testing or osteoporosis medications such as bisphosphonates), and a graph to plot the patient's 10-year risk for fractures. BestPROMPT was designed for physicians to be used at the point of care (Figure 2). The third component of the tool is a paper-based, customized osteoporosis educational (COPE) sheet, which outlines the patient's osteoporosis risks according to their RAQ responses and osteoporosis information pertaining to these identified risks. The COPE sheet can be given to patients to take home at the end of their physician visit (Figure 3). The objectives of the current study were to qualitatively explore how physicians perceive the meaning of CDSSs, the facilitators and barriers to using CDSSs in their own practice; and to determine which critical features, functions, and evidence are needed to transform the conceptual model of the osteoporosis tool into a functional prototype.

## Methods

### Design and sampling

We conducted a qualitative study of focus groups between June and December 2007. The study was approved by the University of Toronto Health Sciences research ethics board. The sampling strategy for the pilot focus group involved randomly selecting family physicians, and specialists involved in the care of patients with osteoporosis from a list of health care providers available from the CPSO (College of Physicians and Surgeons of Ontario) database (representing 13,298 active family physicians and 3520 general internists). We considered for inclusion all full-time physicians practicing in the greater Toronto area who used any type of patient record system. Of 406 faxed invitations, 8 participants agreed to participate and 5 attended (3 family physicians, 2 internists) the pilot focus group. Recruitment involved sending invitations using a faxed letter, which included a demographic questionnaire and consent form. The demographic questionnaire included a modified version of the technology profile inventory (TPI) [26], which is a validated instrument used to measure participants' baseline attitudes toward computers and the Internet according to 3 reliable factors: *Interest, Approval, and Confidence *[26]. To achieve a rigorous qualitative methodology and saturation of themes [27,28], we planned to recruit 5-8 participants per focus group, and to stratify the sample to ensure representation of participants from rural and urban settings, and from university and non-university affiliated sites. To increase our sample for subsequent focus groups, we switched to a convenience sampling strategy where we faxed invitations to the remaining 1324 physicians representing the greater Toronto area, of which 9 family physicians and 2 internists attended 3 additional focus groups.

### Focus group sessions

An experienced moderator facilitated the discussion and flow of the focus groups using a structured interview guide (see additional file 1). The interview guide was pilot tested with 2 family physicians and a general internist to ensure that they were clear and well understood. Each focus group session lasted 1.5 hours and consisted of 2 parts. Part 1 was devoted to generating discussion about participants' baseline understanding of CDSSs, and to explore their perception of the facilitators and barriers to using CDSSs in the context of their own practice. We collected these baseline perceptions so that participants could provide unbiased feedback about CDSSs prior to being introduced to the conceptual osteoporosis tool. In Part 2, participants provided comments about the tool without any prompts from the moderator.

### Data collection and analysis

Focus group sessions were audiotaped and transcribed verbatim. Data collection and quantitative content analyses were guided by the constant comparative method of grounded theory methodology [27]. We used this methodology because it facilitates the understanding of a phenomenon that has not previously been studied (i.e. the conceptual osteoporosis tool), and it enables the exploration of the ways in which the "reality" of the tool is socially constructed [29], particularly to clarify how the tool might be used at the point of care in real practice settings.

Data was coded from transcripts using a process of open, axial and selective coding [28,29] using NVivo 8 software (QSR International, 2008). Two investigators (MK, CM) independently developed a coding scheme by identifying, classifying, and labelling the primary patterns in the content. During open coding, the constant comparative approach was used to group the codes into categories (where each category was considered as a unit of analysis) and identified themes. Axial coding was then applied to look at the inter-relationship of categories [29]. The frequency and consistency in which participants indicated categories in the transcripts was used to provide credibility to these categories. Inter-coder reliability between the 2 investigators was assessed using Kappa statistics (in NVivo 8), and any disagreements (considered as < 90% agreement) were resolved by consensus by a third investigator (SES).

Analysis involved a continuous iterative process, whereby data from the pilot focus group were re-examined, and identified concepts were explored in the subsequent focus groups. The analysis was thus cumulative and iterative, with each focus group building on the discussions of the proceeding group (e.g. focus group interview questions and components of the osteoporosis tool design were modified and refined for transcripts of subsequent focus groups) until themes were saturated.

## Results

The characteristics of 16 participants (12 family physicians, 3 general internists, and 1 rheumatologist) from 4 focus groups are in Table 1. Seventy-five percent of participants were family physicians and men, and practicing for > 25 years (44%), in mostly urban or inner city centres (56%). Most physicians (87%) practiced in a private office or clinic setting, and were in a group or combination of group and academic type of practice (56%). Of the proportion of participants who utilized an electronic health record (EHR) or computer physician order entry (CPOE) system in their practice (31-37%), the range of integration of these systems varied widely (< 10% to 100%). The TPI score indicated that 87% of participants had a positive attitude toward computers and the Internet (average TPI score 3.8) (Table 2).

**Table 1:** Characteristics of focus group participants (N = 16)*

Characteristic	N (%)
**Gender**	

Men	12 (75)

Women	4 (25)

**Age range (years**)	

25-35	2 (12)

36-45	4 (25)

46-55	2 (12)

56-65	6 (37)

> 65	2 (12)

**Type of physician**	

Family	12 (75)

General Internal Medicine	3 (19)

Other specialist: Rheumatology	1 (6)

**Years in practice (years)**	

< 5	1 (6)

5-10	2 (12)

11-15	3 (19)

16-25	3 (19)

> 25	7 (44)

**Type of practice**	

Group	7 (44)

Solo	4 (25)

Academic	2 (12)

Combination	3 (19)

**Practice setting**	

Private office/clinic	14 (87)

Academic centre	3 (19)

**Practice location**	

Urban	9 (56)

Inner city	6 (37)

Suburban	1 (6)

**Using an EHR or CPOE**	

EHR (range of integration: partial to 99%)	6 (37)

CPOE (range of integration: < 10% to 100%)	5 (31)

*EHR = electronic health record; CPOE = computerized provider order entry.

**Table 2:** Participants' attitudes toward computers and the Internet (N = 15)*

Technology Profile Inventory (TPI) Factor	Average TPI score†
Interest	3.6

Approval	4.5

Confidence	3.6

**Average TPI score across all factors**	**3.8**

*Adapted from Spence I, DeYoung CG, and Feng J. The Technology profile inventory: Construction, validation, and application *Computers in Human Behaviour *2009, 25(2):458-465. †Score is based on a 5-point Likert scale where 1 = strongly disagree, 3 = neutral, 5 = strongly agree.

We identified 3 broad categories of themes: 1) Participants' perception and understanding of the meaning and use of CDSSs; 2) Participants' identification of problems with the RAQ component of the osteoporosis tool, and suggestions for modifying specific questions; and 3) The facilitators and barriers to using the 3 components of the tool.

### 1) Perception and understanding the meaning, and the use of CDSSs

We identified 5 themes from participants' description of CDSSs:

#### Theme 1: The perception and understanding of the meaning of CDSSs

Participants understood the meaning of CDSSs as a *"device or system or program or guideline that takes them down a pathway that helps to decide on an appropriate decision given certain parameters of patients"*. Most described it as *"a tool where you can plug in data"*, *"an algorithm that can be used in a computer system"*, or a tool that *"asks for patient data and provides case-specific advice on how to proceed"*. Some participants described CDSSs in the context of risk assessment at the point of care: *"For me a clinical decision support tool would be for example a test, a rapid screening test that helps us to make a quick diagnosis so you can treat it on the spot..."*. Most participants expressed that CDSSs should work consistently, be evidence-based, and provide a level of standardization to every day practice.

#### Theme 2: Format, characteristics, and features of CDSSs

Participants described CDSSs within a paper-based context such as laminated cards and questionnaires with "tick boxes", but most thought that the format should be computer-based using a handheld device or a Tablet PC. Several participants described the use of a flowchart-based system such as the "Framingham" cardiovascular risk assessment tool [30], which was frequently cited as an example to describe CDSS. Positive features of CDSSs were described as quick to use, user-friendly (simple, clear, easy to use), accessible (portable, small), and inexpensive. Negative features were described as confusing, cumbersome or difficult to use, and not accessible or portable.

#### Theme 3: Components to include, and data processing and navigation of CDSSs

Participants suggested that CDSSs should include reminders for appropriate diagnosis and treatment, major and minor risk factors, a 10-year fracture risk graph, lab tests, an option for disease management strategies, and a search box for evidence-based information. Most thought that CDSSs should be algorithm and evidence-based, problem centered, and be able to generate something that can be printed and given to patients. Having too many choices, or layered links or pathways were identified as barriers to use: "*I don't want to go through 12 different layers before I finally get to things."*

#### Theme 4: When and where to use CDSSs

Most participants indicated that they would use a CDSS at the point of care, but only if the system did not take too much time to use: *"If it is going to be something that takes 10 minutes to go through from start to finish then it takes too much time." *Suggestions were to use CDSS during a physical examination appointment or following the patient visit if the problem was too complex, and to administer the RAQ component of the tool in the reception area or examination rooms if patients were involved in completing the questionnaire. However, they pointed out that barriers in some settings might be the lack of space in exam rooms or lack of confidentiality in the reception area.

#### Theme 5: Preparation before a patient visit

Most participants do not have time to prepare before a patient visit and have little time between visits (range between 10 seconds to a few minutes). Most described their preparation for a visit as reviewing patient charts while walking the patient to the examination room or reviewing the chart in front of the patient during the visit. Assistance from nurses or clinic staff in the form of notes and reminders on the chart (e.g. the reason for the visit, if the visit is a physical appointment, things to do for the next visit, abnormal lab or radiology results, and other results of completed tests) was identified as a major facilitator for preparation before patient visits.

### 2) Problems with the Risk Assessment Questionnaire (RAQ), and suggestions for modifying questions

**Figure 4:**
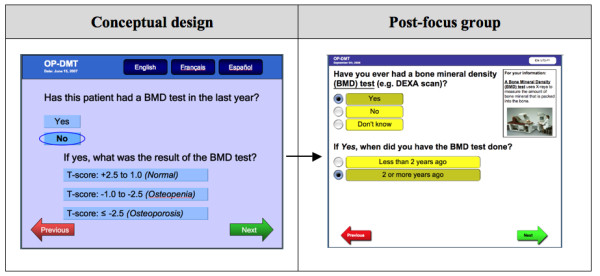
**RAQ question about bone mineral density testing**.

Participants identified problems with 7 of the 13 questions in the RAQ, of which 4 questions required the most extensive modifications: First, most focus group participants thought that clinic staff would not have time to answer the question about BMD test results (i.e. to extract T-scores) (Figure 4). Additionally, they were concerned that extracting and recording T-scores requires interpretive skills and thus training, which could further burden time and available resources: *"I don't think pulling BMD test results from patient charts will happen in our setting...I mean no one has time to do that... also should the admin person be looking at the results, unless they knew what to look at, and how would they interpret it?"*. Although the extractability of BMD test results was important for the design of the RAQ because T-scores can be used to predict osteoporosis and fracture risk [31], this question was consequently modified to redirect the question to patients, but to probe only for information on whether or not they have ever had a BMD test, and if yes, whether it was done within or over 2 years ago.

**Figure 5:**
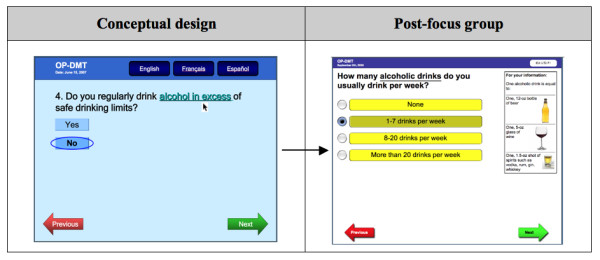
**RAQ question about alcohol consumption**.

Second, participants disagreed with the wording of the caffeine and alcohol questions (Figure 5) because they believed that patients would not respond honestly: *"Patients lie to you. You have to ask the question in a different way so they don't get threatened"*. They also thought that the term: "drinking in excess" may be confusing for patients. Participants suggested providing a wider selection of response options consisting of varying amounts of alcohol or caffeine consumed (or none), and to ask the question in terms of weekly rather than daily consumption for alcohol. Other suggestions were to provide definitions and pictures for alcohol and caffeine units.

**Figure 6:**
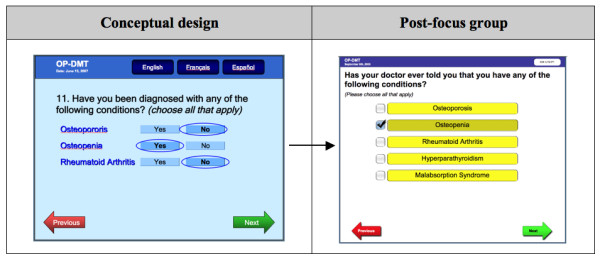
**RAQ question about medications**.

Third, participants were concerned that patients might not recognize or understand "osteopenia" or may be confused about the term: "rheumatoid arthritis" in the question probing for conditions (Figure 6). Participants suggested rephrasing the question to: "*have you ever been told by a physician that you have one of these conditions?" *because *"chances are they don't have the condition if they never heard of it."*

**Figure 7:**
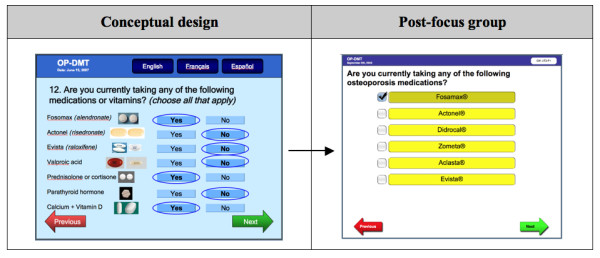
**RAQ question about conditions**.

In the fourth question requiring extensive modifications, participants thought that it was a good idea to show pictures of medications (Figure 7), but did not think that patients would recognize pictures of pills. They also identified several other osteoporosis medications that should be added to the list such etidronate (e.g. *Didrocal^®^*) and anti-seizure medications to the list such as phenytoin (e.g. *Dilantin^®^*) and carbamazepine (e.g. *Tegretol^®^*). Other suggestions were to represent the medications in categories, and to ensure that the list is continuously updated as guidelines change.

### 3) Facilitators and barriers to using the 3 components of the osteoporosis tool (ie, the RAQ, BestPROMPT and COPE)

#### Component 1: Risk Assessment Questionnaire (RAQ)

Participants were concerned about confidentiality and possible damage to the Tablet PCs if patients completed the RAQ in the waiting room/reception area. Suggestions to overcome these problems were to complete the questionnaire in the examination rooms, which would provide more security for the Tablet PCs and more privacy for patients. Others suggested that an appointment dedicated to osteoporosis or a physical examination visit might provide more time for patients to complete the RAQ. Participants were also concerned that patients might not understand the RAQ questions or provide inaccurate responses, particularly elderly patients or those with limited or no computer experience. Suggestions for improving the RAQ were to organize the questions in the order of risk factors as outlined in guidelines, to include a "Warning" if patients missed a question, and to add an introduction about osteoporosis at the beginning of the questionnaire.

#### Component 2: Best practice recommendation prompt (BestPROMPT) for physicians

Participants liked the BestPROMPT output, and considered the section outlining the major and minor risk factors for osteoporosis as the most helpful. Identified barriers were related to content and usability of the BestPROMPT. For example, many participants were not familiar with or did not find the osteoporosis risk assessment instrument (ORAI, which can help identify women who should have a BMD test) [32] as a value-added feature for determining who should receive a BMD test. Suggestions for improving the BestPROMPT were to add a section on "lifestyle" such as physical activity, smoking, and diet as part of the suggested treatment recommendations, and to provide something "visual" such as a 10-year risk graph to help patients conceptualize their fracture risk.

Most participants indicated that they would use the sheet in front of their patient at the point of care, but also pointed out that lack of time could be the largest barrier: *"You see 50 people a day, so even if it takes a minute, that is another hour a day. I don't want to stay another hour just to screen for osteoporosis"*. Although physicians understood the benefits of the BestPROMPT, they did not think it was feasible to generate the sheet for every patient or every visit.

#### Component 3: Customized osteoporosis education (COPE) for patients

Participants believed that the COPE sheet would provide useful information to patients about osteoporosis: *"It would be a very strong tool for somebody to come out of an office with a thing saying my risk factors are this and my diagnosis is this and I am supposed to do this. That would be very, very powerful." *However, participants emphasized that the wording of the COPE sheet needs to be more balanced so that patients don't stop taking important medications for other conditions that are associated with osteoporosis risk: *"You've got to be very careful with what you say in the patient printout. You're actually saying that if you're taking prednisone this will harm your bone density. Then she's going to stop prednisone and her asthma is going to become terrible. So, you have to be very careful in what you say to patients because you don't want them to stop taking their asthma medication."*

Other suggestions were to increase the print size, and to include statements that encourage patients to discuss the suggested treatment recommendations with their physician.

## Discussion

**Figure 8:**
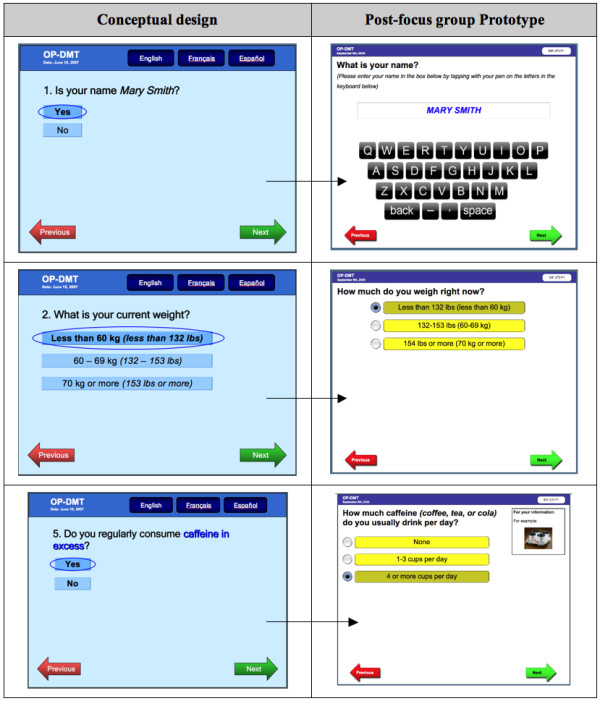
**Screen shots of the evolution of selected Risk Assessment Questionnaire (RAQ)**.

**Figure 9:**
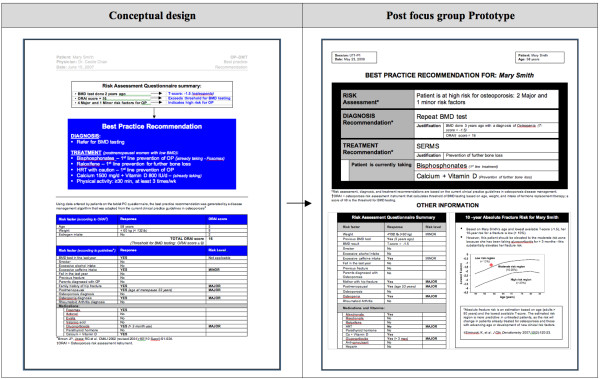
**Screen shot of the evolution of the Best Practice Recommendation Prompt (BestPROMPT) sheet**.

**Figure 10:**
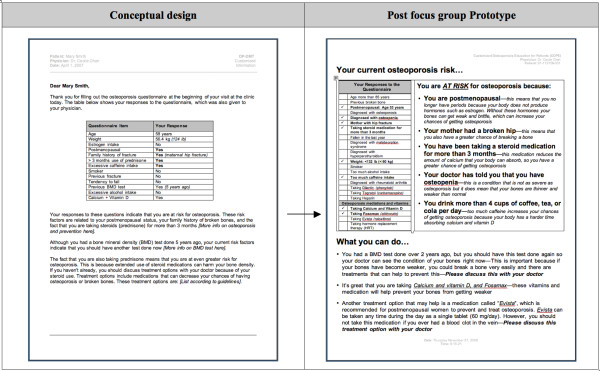
**Screen shot of the evolution of the Customized Osteoporosis Education (COPE) sheet)**.

Our study revealed physicians' understanding of CDSSs in the context of what they would find useful in their own practice. The progressive analysis and iterative focus group structure was useful for identifying the specific features and functions that physicians perceived as important to include in CDSSs--to be computer-based, user-friendly, to enhance workflow, and to be accessible at the point of care. The finding that "lack of time" was perceived as the largest barrier to using CDSSs is not surprising, as this has been shown consistently in previous studies [33-35], and hence should be an important consideration when designing CDSSs. The time burdens on physicians just before and during a clinical encounter confirmed our decision to design the tool to target the completion of the RAQ to patients. Potential solutions to meet this challenge include ensuring that the risk assessment component of the tool could be used without assistance and within a short period of time (e.g. 5 minutes) by the target patient population (i.e. men ≥ 65 years of age and postmenopausal women), and that the physician component of the tool was also quick to use, clear, and concise. Our study also revealed the specific facilitators and barriers to the use of each of the 3 components of the conceptual osteoporosis tool. These findings were useful for informing the transformation of the conceptual design of the osteoporosis into a functional prototype, but can also be relevant to the development of other, similar tools. Please see Figures 8, 9, 10 for screenshots of the evolution of the 3 components of the osteoporosis tool from conceptual design to post-focus group prototype.

During our exploration of themes, several important challenges emerged, which will be considered when the prototype is further refined in usability and evaluation studies. Evidence indicates that CDSSs can be programmed within EHR systems, have the potential to successfully facilitate the delivery of evidence-based, patient-specific decision support at the point of care, and improve guideline adherence [36-38]. The conceptual model of our osteoporosis tool was designed with this type of integration so that risk assessment data could be directly extracted from the EHR. This was important in our design because the time it takes to use the tool can be greatly reduced if fewer pieces of information are entered into the RAQ. The integration between CDSSs and EHRs also facilitates the components of clinical decision support (for physicians) or delivery of education (for patients) to be available at the point of care. However, in the US and Canada, this type of integration may be a challenge for several reasons.

First, the adoption of EHR systems overall remains low at less than 30% in North America [39-41] compared with other developed countries such as Australia, Norway, and the UK that report EHR adoption rates of up to 60-90% [42]. The current study found usage was slightly above the US and Canada rates (31-37%), but the level of EHR integration varied widely (< 10% to 100%). These results are consistent with a recent national survey in the US, which showed that only 4% of survey respondents were fully integrated with an EHR system and only 13% used a basic system [40]. This implies that adoption does not guarantee that physicians will use EHRs for all practice functions.

Second, there are an overwhelming number of EHR vendors available in the US (> 250) and Canada (>45) [43,44]. These systems vary widely in features and capabilities, most are independently owned, and most use proprietary non-standards-based integration interfaces, which makes integration with other software difficult [45]. Wide-scale implementation of CDSSs would then be a challenge, even if EHR systems were in place, because the vendors differ in their protocols and standards for accepting third-party software programming. The slow progress toward the development of interoperable systems also contributes to the problem [46,47]. Although the inability to integrate CDSSs with EHR systems may diminish their potential [37], this does not decrease their potential value for improving outcomes--CDSSs can still be developed and used as standalone systems to positively impact clinical practice.

Our data also revealed that workflow differences and the role of customization are important factors that need to be considered during the tool design process. Although workflow analysis techniques are often used prior to implementing hospital health information systems, they have largely been neglected in small practice settings even though this is where the majority of patient care is provided [48]. A systematic review of CDSSs found that only 13% of trials evaluated the impact of the CDSS on clinician workflow [37]. The lack of workflow analysis in small physician practices may also in part explain low EHR and CDSS adoption rates and tool implementation failures. When asked to comment on the conceptual design of the tool during the focus group sessions, the conflicting barriers and facilitators that were identified for the osteoporosis tool clearly exposed the complexity of family physicians' practice settings and their widely differing practice workflows. For example, the success of the point-of-care feature of the osteoporosis tool (i.e., the delivery of the BestPROMPT sheet for physicians just before they see their patients) is directly dependent on the processes that are used by individual physicians to prepare for a patient visit. In the focus group study, this ranged from taking a few minutes to look at the EHR, to reviewing a paper chart on the way to the examination room. This practice variability has major implications on the intended function of the tool to deliver practice recommendations at the point of care. To meet the specific needs of physicians, customization of information technology systems such as the osteoporosis tool may be needed by matching and supporting the desired workflow [48].

### Limitations

There are a number of limitations to our study that are related to the inherent challenges to conducting qualitative studies. We attempted to minimize selection bias by randomly selecting participants from a homogeneous group of physicians for the pilot focus group. However, it was not possible to continue random selection for subsequent focus groups because the response rates were low so we adjusted our sampling strategy to be purposive. Although generalizability may have been affected, it is possible to "transfer" findings to other environments by taking into consideration how well they fit with the current study's methods, procedures, and audience [27].

Another potential limitation was that we did not plan focus groups with patients at risk for osteoporosis. As this focus group study represented the early stages of the tool development process, patients were excluded because it was important to first understand the CDSS needs of physicians and how the conceptual osteoporosis tool might fit into their practice and workflow. We planned a priori, to use this information to then inform the level of involvement that would be needed by patients for the development of the risk assessment component of the tool.

We addressed other potential threats to validity by pilot testing the focus group questions to ensure that they were well understood, and to use an experienced moderator to lead focus group discussions. To limit potential biases that may be introduced by investigators, we standardized procedures, methods, and analysis strategies across all 4 focus groups. Sessions were planned so that physicians were prompted about their perceptions of CDSSs before introducing the conceptual design of the osteoporosis tool. Lastly, we sent participants a summary report of the focus group sessions at the end of the study to provide an opportunity to verify the content of the discussions.

### Next steps

Using study findings and consultation with information technologists and human factors engineers, the conceptual design of the tool was transformed into a working prototype. Evaluation of the prototype will begin with usability testing of the tool on all end users (i.e. physicians and patients) using the method described by Kushniruk *et al *[49]. Usability evaluation of the tool is an important step to avoid problems and errors, which can occur if the needs of end users are not considered as part of the tool development process [50]. Once the osteoporosis tool is further refined during usability evaluation, the prototype will be implemented in 3 family practice settings and pilot tested in an evaluation study.

## Conclusions

Findings from our progressive focus groups were used to develop a functional prototype that may aid physicians in their clinical decision making in osteoporosis disease management at the point of care. The prototype incorporates all aspects of disease management (risk assessment, diagnosis, and treatment), and is multi-targeted to deliver clinical decision support for physicians and education for patients about osteoporosis.

## Competing interests

The authors declare that they have no competing interests.

## Authors' contributions

All authors participated in the design of the study. MK and CM conducted the focus groups. MK, CM, and SES performed the analysis. MK drafted the manuscript, and all authors read and approved the final manuscript.

## Pre-publication history

The pre-publication history for this paper can be accessed here:

http://www.biomedcentral.com/1472-6947/10/40/prepub

## Supplementary Material

Additional file 1 **Focus group interview guide**. Semi-structured questions that was used in the focus groups with physicians. Click here for file

## References

[B1] Gullberg B Johnell O Kanis JA Osteoporosis Int 1997 7 407 13 10.1007/PL00004148 9425497

[B2] Lane NE Epidemiology, etiology, and diagnosis of osteoporosis Am J Obstet Gynecol 2006 194 S3 11 10.1016/j.ajog.2005.08.047 16448873

[B3] National Osteoporosis Society http://www.nos.org.uk/Accessed on April 10, 2009.

[B4] Burge R Dawson-Hughes B Solomon DH Wong JB King A Tosteson A Incidence and Economic Burden of Osteoporosis-Related Fractures in the United States, 2005-2025 J Bone Min Res 2007 22 3 465 75 10.1359/jbmr.061113 17144789

[B5] Osteoporosis Canada http://www.osteoporosis.ca/english/home/Accessed on February 23, 2007.

[B6] Goeree R Blackhouse G Adachi J Cost-effectiveness of alternative treatments for women with osteoporosis in Canada Curr Med Res Opin 2006 22 7 1425 1436 10.1185/030079906X115568 16834841

[B7] Osteoporosis prevention, diagnosis and therapy. NIH consensus statements 2000 17 1 1 45 11525451

[B8] International Osteoporosis Foundation http://www.iofbonehealth.org/facts-and-statistics.html Accessed on April 10, 2009

[B9] Reginster J Burlet N Osteoporosis: A still increasing prevalence Bone 2006 38 S4 S9 10.1016/j.bone.2005.11.024 16455317

[B10] Lips P van Schoor NM Quality of life in patients with osteoporosis Osteoporosis Int 2005 16 447 455 10.1007/s00198-004-1762-7 15609073

[B11] Cummings SR Melton LJ Epidemiology and outcomes of osteoporotic fractures Lancet 2002 359 1761 7 10.1016/S0140-6736(02)08657-9 12049882

[B12] Brown, JP Josse RG for the Scientific Advisory Council of the Osteoporosis Society of Canada 2002 clinical practice guidelines for the diagnosis and management of osteoporosis in Canada (revised, August 26, 2004) CMAJ 2002 167 10 S1 S34 12427685 PMC134653

[B13] Osteoporosis, Clinical Guidelines for Prevention and Treatment: Update on pharmacological interventions and algorithm for management 2003 Royal College of Physicians, Bone and Tooth Society of Great Britain http://www.rcplondon.ac.uk Accessed on April 10, 2009.

[B14] ACOG Committee on Practice Bulletins ACOG Practice Bulletin. Clinical Management Guidelines for Obstetrician-Gynecologists. Number 50, January 2004. Osteoporosis Obstet Gynecol 2004 103 1 203 16 14704265

[B15] Papaioannou A Giangregorio L Kvern B Boulos P Ioannidis G Adachi JD The osteoporosis care gap in Canada BMC Musculoskelet Disord 2004 6 5 11 10.1186/1471-2474-5-11 PMC420244 15068488

[B16] Cheng N Green ME Osteoporosis screening for men: Are family physicians following guidelines? Can Fam Phys 2008 54 1140 1.e1-5 PMC2515229 18697977

[B17] Jaglal SB McIsaac WJ Hawker G Carroll J Jaakkimainen L Cadarette SM Cameron C Davis D Information needs in the management of osteoporosis in family practice: an illustration of the failure of the current guideline implementation process Osteoporosis Int 2003 14 672 6 10.1007/s00198-003-1421-4 12879224

[B18] Grimshaw JM Thomas RE MacLennan G Fraser C Ramsay CR Vale L Whitty P Eccles MP Matowe L Shirran L Wensing M Dijkstra R Donaldson C Effectiveness and efficiency of guideline dissemination and implementation strategies Health Technology Assessment 2004 8 6 1 72 iii-iv 10.3310/hta8060 14960256

[B19] Randolph AG Haynes RB Wyatt JC Cook DJ Guyatt GH Users' guides to the medical literature XVII. How to use an article evaluating the clinical impact of a computer-based clinical decision support system JAMA 1999 281 67 74 10.1001/jama.282.1.67 10404914

[B20] Bates DW Kuperman GJ Wang S Gandhi T Kittler A Volk L Spurr C Khorasani R Tanasijevic M Middleton B Ten commandments for effective clinical decision support: Making the practice of evidence-based medicine a reality JAMIA 2003 10 6 523 530 12925543 10.1197/jamia.M1370 PMC264429

[B21] Purcell GP What makes a good clinical decision support system: We have some answers, but implementing good decision support is still hard BMJ 2005 330 741 2 10.1136/bmj.330.7494.740 15802700 PMC555864

[B22] Sullivan F Wyatt JC How decision support tools help define clinical problems BMJ 2005 331 831 833 10.1136/bmj.331.7520.831 16210284 PMC1246087

[B23] Kastner M Straus SE Clinical decision support tools for osteoporosis disease management: A systematic review of randomized controlled trials JGIM 2008 23 12 2095 2105 10.1007/s11606-008-0812-9 18836782 PMC2596508

[B24] Cranney A Lam M Ruhland L Brison R Godwin M Harrison MM Harrison MB Anastassiades T Grimshaw JM Graham ID A multifaceted intervention to improve treatment of osteoporosis in postmenopausal women with wrist fractures: a cluster randomized trial Osteoporosis Int 2008 19 1733 40 10.1007/s00198-008-0669-0 18629567

[B25] Kanis JA on behalf of the World Health Organization Scientific Group Assessment of osteoporosis at the primary health-care level. Technical Report 2008 WHO Collaborating Centre, University of Sheffield, UK

[B26] Spence I DeYoung CG Feng J The technology profile inventory: Construction, validation, and application Computers in Human Behaviour 2009 25 2 458 465 10.1016/j.chb.2008.10.009

[B27] Krueger RA Casey MA Focus Groups: A practical guide for applied research 2000 3 California: Sage Publications Inc.

[B28] Patton MQ Qualitative Research and Evaluation Methods 2002 3 California: Sage Publications Inc.

[B29] Strauss AL Corbin JM Basics of qualitative research: Techniques and procedures for developing grounded theory 1998 California: Sage Publications Inc.

[B30] Wilson PW D'Agostino RB Levy D Belanger AM Silbershatz H Kannel WB Prediction of coronary heart disease using risk factor categories Circulation 1998 97 1837 1847 9603539 10.1161/01.cir.97.18.1837

[B31] Siminoski K Leslie WD Frame H Hodsman A Josse RG Khan A Lentle BC Levesque J Lyons DJ Tarulli G Brown JP Recommendations for bone mineral density reporting in Canada: A shift to absolute fracture risk assessment J Clin Densitometry 2007 10 2 120 23 10.1016/j.jocd.2007.01.001 17485028

[B32] Cadarette SM Jaglal SB Kreiger N McIsaac WJ Darlington GA Tu JV Development and validation of the Osteoporosis Risk Assessment Instrument to facilitate selection of women for bone densitometry CMAJ 2000 162 9 1289 1294 10813010 PMC1232411

[B33] Legare F Ratte S Gravel K Graham ID Barriers and facilitators to implementing shared decision-making in clinical practice: Update of a systematic review of health professionals' perceptions Patient Ed and Counselling 2008 73 526 35 10.1016/j.pec.2008.07.018 18752915

[B34] Short D Frischer M Bashford J Barriers to the adoption of computerized decision support systems in general practice consultations: a qualitative study of GPs perspectives Int J Med Inform 2004 73 357 362 15135754 10.1016/j.ijmedinf.2004.02.001

[B35] Sullivan F Mitchell E Has general practitioner computing made a difference to patient care? A systematic review of published reports BMJ 1995 311 848 52 7580494 10.1136/bmj.311.7009.848 PMC2550856

[B36] Wells S Furness S Rafter N Horn E Whittaker R Stewart A Moodabe K Roseman P Selak V Bramley D Jackson R Integrated electronic decision support increases cardiovascular disease risk assessment four fold in routine primary care practice Eur J Cardiovasc Prev and Rehab 2008 15 173 178 10.1097/HJR.0b013e3282f13af4 18391644

[B37] Garg AX Adhikari NKJ McDonald H Rosas-Arellano MP Devereaux PJ Beyene J Sam J Haynes RB Effects of computerized clinical decision support systems on practitioner performance and patient outcomes: A Systematic Review JAMA 2005 293 10 1223 1238 10.1001/jama.293.10.1223 15755945

[B38] Kwok R Dinh M Dinh D Chu M Improving adherence to asthma clinical guidelines and discharge documentation from emergency departments: Implementation of a dynamic and integrated electronic decision support system Emerg Med Australia 2009 21 31 37 10.1111/j.1742-6723.2008.01149.x 19254310

[B39] Anderson GF Frogner BK Johns RA Reinhardt UE Health care spending and use of information technology in OECD countries Health Aff (Milwood) 2006 25 3 819 31 10.1377/hlthaff.25.3.819 16684749

[B40] DesRoches CM Campbell EG Rao SR Donelan K Ferris TG Jha A Kaushal R Levy DE Rosenbaum S Shields AE Blumenthal D Electronic health records in ambulatory care - A national survey of physicians NEJM 2008 359 1 50 60 10.1056/NEJMsa0802005 18565855

[B41] Mitiku TF TU K ICES Report: Using data from electronic medical records: Theory vs practice Healthcare Quarterly 2008 11 4 23 25 19066478 10.12927/hcq.2008.20088

[B42] Protti D A comparison of information technology in general practice in ten countries Health Q 2007 10 107 16 17491575

[B43] Leavitt J Selecting an Electronic Medical Record (EMR) for a Gastroenterology Practice Am J Gastroenterol 2008 103 2423 27 10.1111/j.1572-0241.2008.02058.x 18855851

[B44] Canadian EMR: EMR vendors http://www.canadianemr.ca/index.aspx?PID=13 Accessed on April 13, 2009

[B45] Moore BJ Gaehde S Curtis C Architectural choices and challenges of integrating electronic patient questionnaires into the electronic medical record to support patient-centred care AMIA Proc 2008 490 4 PMC2655980 18999024

[B46] American Health Information Management Association and American Medical Informatics Association Terminology and Classification Policy Task Force. Healthcare Terminologies and Classifications: Essential keys to interoperability http://library.ahima.org/xpedio/groups/public/documents/ahima/bok1_034273.pdf

[B47] Brailer DJ Interoperability: the key to the future of the health care system Health Aff 2005 Suppl w5-19 w5-21 10.1377/hlthaff.w5.19 15659454

[B48] Lee J Cain C Young S Chockley N Burstin H The Adoption Gap: health information technology in small physician practices. Understanding office workflow can help realize the promise of technology Health Affairs 2005 24 5 1364 66 10.1377/hlthaff.24.5.1364 16162585

[B49] Kushniruk AW Patel VL Cognitive and usability engineering methods for the evaluation of clinical information systems J Biomed Inform 2004 37 1 56 76 10.1016/j.jbi.2004.01.003 15016386

[B50] Kushniruk AW Triola M Stein B Borycki E Kannry J The relationship of usability to medical error: An evaluation of errors associated with usability problems in the use of a handhald application for prescribing medications Medinfo 2004 11 Pt2 1073 1076 15360977

