# Supplementary figures and images for: Development of a prototype clinical decision support tool for osteoporosis disease management: a qualitative study of focus groups
Source: BMC Med Inform Decis Mak. 2010 Jul 22;10:40. doi: 10.1186/1472-6947-10-40 (PMC2914714; doi:10.1186/1472-6947-10-40)

**Appendix A**

Focus Group Interview Guide


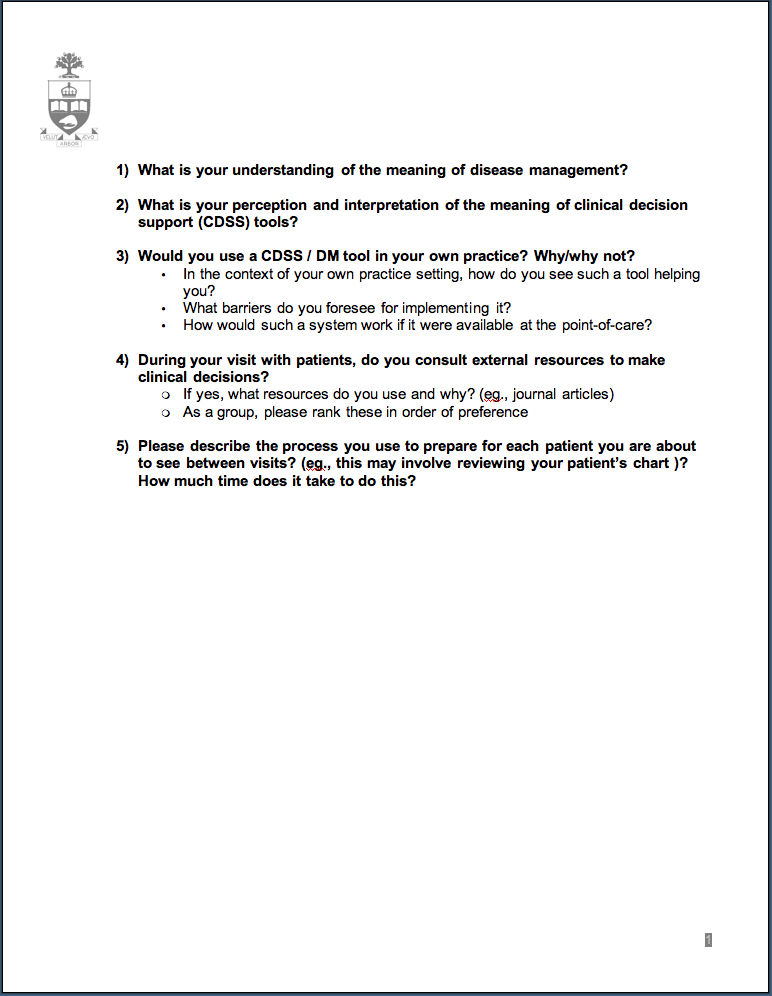

Supplement: Additional file 1 — Focus group interview guide. Semi-structured questions that was used in the focus groups with physicians. [file 1472-6947-10-40-S1.doc]
